# Avulsion péno-scrotale par encornement de zebu

**DOI:** 10.11604/pamj.2015.20.91.5832

**Published:** 2015-02-02

**Authors:** Dera Andraina Ratsimandresy, Auberlin Rakototiana

**Affiliations:** 1 Service d'Urologie, HU Joseph Ravoahangy Andrianavalona, Antananarivo 101 Madagascar

**Keywords:** Avulsion cutanée, encornement, Zébu, lanmbeau, skin avulsion, horn trauma, Zebu, lambeau

## Image en medicine

Le traumatisme des organes génitaux externes chez l'homme est rare comparé aux autres traumatismes du corps humain. Les pertes de substances cutanées péno-scrotales découlent souvent des morsures d'animaux, des traumatismes par armes à feu ainsi que des lésions causées par les machines industrielles. Cette perte de substance met en jeu le pronostic vital mais surtout fonctionnelle du patient. Le cas d'un homme de 40 ans, admit dans notre hôpital pour une avulsion complète de la peau péno-scrotale, de grade V de l'AAST, après encrornement de zébu est rapporté (A). Une plastie complète de recouvrement était réalisée après débridement et nettoyage de la plaie (B). Les suites opératoires étaient simples. Quoique rare, la prise en charge des traumatismes des OGE par encornement de zébu doivent faire parties des palettes du chirurgien de notre pays étant donné que Madagascar compte plus de zébu que de population. Le traitement est difficile et longue. La rapidité de la prise en charge dicte la réussite du traitement.

**Figure 1:**
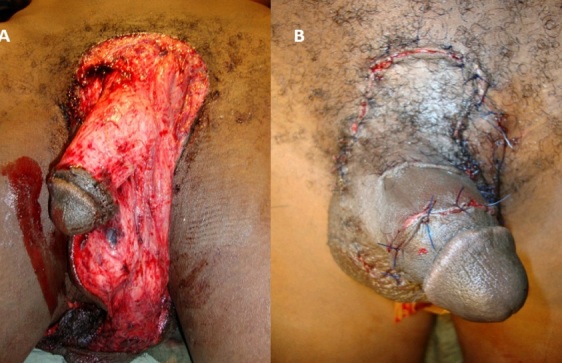
Avulsion péno-scrotale par encornement de zébu

